# Transcriptome sequencing reveals high-salt diet-induced abnormal liver metabolic pathways in mice

**DOI:** 10.1186/s12876-021-01912-4

**Published:** 2021-08-28

**Authors:** Yanping Li, Yufei Lyu, Jing Huang, Kun Huang, Jiufei Yu

**Affiliations:** 1 Department of Gastroenterology, Civil Aviation General Hospital, No. 1, Gaojingjia, Chaoyang District, China; 2 Beijing Institute of Biotecnology, No. 20, Dongda Street, Fengtai District, Beijing, China

**Keywords:** High-salt diet, Transcriptome sequencing, Liver

## Abstract

**Background:**

Although salt plays an important role in maintaining the normal physiological metabolism of the human body, many abnormalities in the liver caused by a high-salt diet, especially with normal pathological results, are not well characterized.

**Methods:**

Eight-week-old female C57BL/6 mice were randomly divided into a normal group and a high salt group. These groups were then fed with normal or sodium-rich chow (containing 6% NaCl) for 6 weeks. Liver injury was evaluated, and the influences of a high-salt diet on the liver were analyzed by transcriptome sequencing at the end of week 6.

**Results:**

We found that although no liver parenchymal injury could be found after high-salt feeding, many metabolic abnormalities had formed based on transcriptome sequencing results. GO and KEGG enrichment analyses of differentially expressed genes revealed that at least 15 enzymatic activities and the metabolism of multiple substances were affected by a high-salt diet. Moreover, a variety of signaling and metabolic pathways, as well as numerous biological functions, were involved in liver dysfunction due to a high-salt diet. This included some known pathways and many novel ones, such as retinol metabolism, linoleic acid metabolism, steroid hormone biosynthesis, and signaling pathways.

**Conclusions:**

A high-salt diet can induce serious abnormal liver metabolic activities in mice at the transcriptional level, although substantial physical damage may not yet be visible. This study, to our knowledge, was the first to reveal the impact of a high-salt diet on the liver at the omics level, and provides theoretical support for potential clinical risk evaluation, pathogenic mechanisms, and drug design for combating liver dysfunction. This study also provides a serious candidate direction for further research on the physiological impacts of high-salt diets.

**Supplementary Information:**

The online version contains supplementary material available at 10.1186/s12876-021-01912-4.

## Background

As an essential mineral in daily life, salt plays an important role in maintaining the normal physiological metabolism of the human body. Although the World Health Organization recommends that the daily intake of salt should not exceed 5 g, the daily intake of salt is often greater than 10 g based on survey data [1]. This is particularly true in some countries and regions where salt intake is often higher due to specific dietary habits [2], which can lead to some potential disease risks in these populations. Studies have found that a long-term high-salt diet results in potential harm to the body, inducing cardiovascular disease [3, 4], insulin resistance and type 2 diabetes [5], metabolic syndrome, obesity, muscle atrophy [6–8], and problems with the immune system [9].

Some mechanisms of organ damage caused by high salt intake have been elucidated thus far. For example, animal experiments have revealed that a high-salt diet can cause kidney damage through the reduction of ACE2, enhancement of leukocyte adhesion, blunt renal autoregulation via a reactive oxygen species-dependent mechanism, etc. [10–12]. A high-salt diet can also cause cardiovascular abnormalities, and high plasma sodium can cause hypertension by directly affecting endothelial functions, thus controlling vascular tone [13], or by participating in cardiovascular injury through hormonal pathways [14]. In addition, a high-salt diet also has direct or indirect effects on the liver and causes diseases like fibrosis and fatty liver [15, 16]. Although many mechanisms of liver damage caused by high-salt diets have been revealed to date, the liver, as the largest metabolic organ, is the site of a variety of important signaling pathways. Whether these are affected by a high-salt diet and are part of the cause of specific diseases is still unclear.

With the development of transcriptome sequencing technology in recent years, it has become feasible to analyze all the mRNAs transcribed by a specific cell or organ in a certain functional state [17–19]. At present, transcriptome sequencing has been applied to many medical fields, such as clinical diagnosis, marker screening, prognosis evaluation, and pathogenesis [20–22].

In this study, we sequenced the liver transcriptome of normal and high-salt diet mice and explored the effects of a high-salt diet on the liver at a gene expression level. In addition to some abnormal signaling and metabolic pathways that have been reported previously, we also identified many new abnormal metabolic pathways, which provides a strong theoretical basis for the potential clinical risks of a high-salt diet, the diseases and pathogenic mechanisms it correlates with, and provides potential targets for a rational drug design to treat high-salt diet-induced liver dysfunction.

## Methods

### Animals

Eight-week-old female C57BL/6 mice, 16–19 g in size, were purchased from Charles River, Beijing, China, and housed in the Laboratory Animal Center of the Academy of Military Medical Sciences, Beijing, China. The mice were randomly divided into a normal group and a high-salt group. The normal group was fed with normal chow (mainly containing crude protein (≥ 18.0%), crude fat (≥ 4.0%), crude fiber (≤ 5.0%), calcium (1.0–1.8%), phosphorus (0.6–1.2%), water (≤ 10.0%), and ash (≤ 8.0%)), and the high-salt group was fed with sodium-rich chow (containing 6% NaCl) for 6 weeks. Analysis was performed at the end of week 6.

### Hematoxylin–eosin staining (H&E staining)

H&E staining was performed according to the instructions of a Hematoxylin–Eosin Staining Kit (Solarbio, China, #G1121). Briefly, after dissection, the liver tissues of mice were fixed with a 4% fixative solution, embedded in paraffin, and then sectioned to about 3 μm in thickness. The paraffin sections were washed twice with xylene for 10 min, and then washed sequentially with absolute ethanol, 95% ethanol, 80% ethanol, 70% ethanol, and distilled water for two minutes. Next, sections were stained with hematoxylin for 5 min and washed with water for 8 s. The sections were then incubated in differentiation solution for 5 s and again washed with water for 30 s. After incubation in blue returning solution for 1 min, the sections were rinsed with water for 30 s and eosin-stained for 1 min. Next, the sections were washed with water, 80% ethanol, 90% ethanol, 95% ethanol, 95% ethanol, absolute ethanol for 5 s each, and then washed with absolute ethanol and xylene for 1 min each. Finally, they were fixed with neutral balata.

### Biochemical tests

Blood samples were taken by tail snip on week 6 and serum was collected after incubating at 4 °C overnight. The detection of alanine aminotransferase (ALT), aspartate transaminase (AST), and alkaline phosphatase (ALP) from serum was performed according to the instructions of ALT, AST, and ALP Detection Kits (Condical, Zhejiang, China, #E10001-5, #E10002-5, and #E10003-5), respectively. The data was collected using a Biochemical Autoanalyzer (TBA-40, Toshiba).

### RNA extraction

After mice were euthanized, liver tissues were removed and bulk RNA was extracted according to the instructions of a Total RNA Extraction Kit (Solarbio, China, #R1200).

### Construction of transcriptome libraries

First, mature mRNAs were isolated from total RNA using oligo (dT) magnetic beads, and these were then randomly fragmented by mixing with fragmentation buffer. Then, first-strand cDNAs were synthesized using mRNAs as templates, and second-strand cDNAs were synthesized by adding polymerase chain reaction (PCR) buffer, dNTPs, RNase H, and DNA polymerase I. After purification, the double-strand cDNAs were harvested with elution buffers for end repair and A-tailing, target fragments were size selected using agarose gel electrophoresis, and final libraries were obtained by PCR amplification. After passing library quality inspection, an Illumina platform was used to carry out high-throughput sequencing.

### Sequencing data quality control

First, the data was filtered by removing contaminating sequences, low-quality sequences, and sequences containing more than 5% n. Then, the mass distribution and base distribution were analyzed and compared to ensure the reliability of subsequent analyses. Finally, the filtered sequences from each sample were compared to a mouse reference genome.

### Statistical analysis

Data is expressed as the mean ± standard deviation, and statistical analyses were completed using GraphPad 8.0. Student’s *t*-test was used to compare the difference between two groups, and differences were considered statistically significant at *p* < 0.05.

## Results

### Effects of a high-salt diet on liver function in mice

**Fig. 1:**
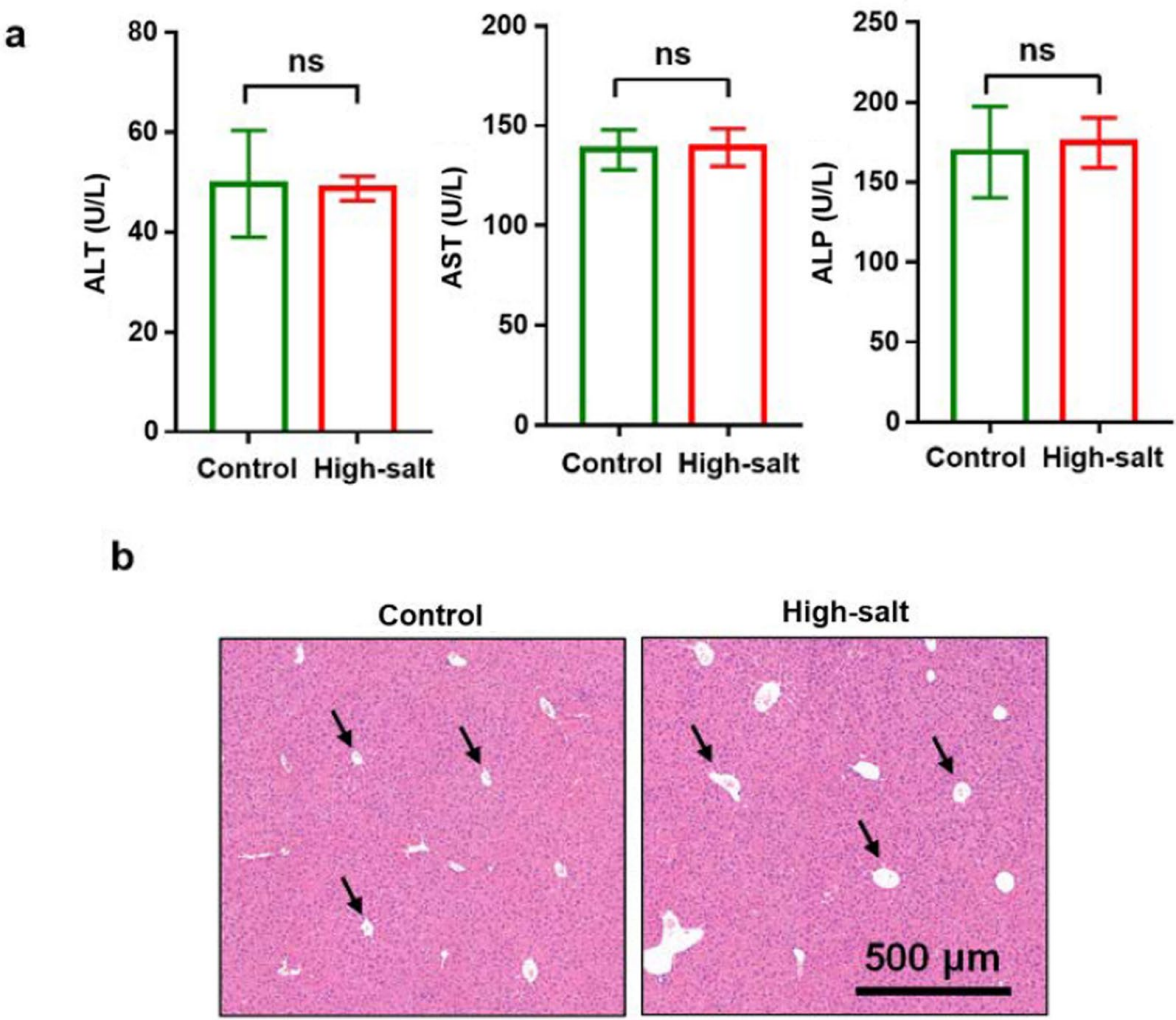
The effect of a high-salt diet on liver function: **a** ALT, AST, and ALP in blood serum after 6 weeks of feeding (n = 5). ns indicates no significant difference; **b** H&E staining of liver tissue after 6 weeks. The arrows point to the central veins of the hepatic lobule

Although there have been many reports on the effects of long-term high-salt diets on the liver, salt, as a common flavoring agent, is ubiquitous in cuisine and may cause this damage to be very chronic and long-term over an average human’s lifespan. In order to evaluate whether liver function could be affected by high-salt (6% NaCl) diet, biochemical indices including ALT, AST, and ALP in blood serum were detected after 6 weeks of feeding mice a high-salt diet. Results showed that there were no significant differences in these biochemical indices between normal and high-salt diet mice (Fig. 1a). Moreover, H&E staining of liver tissue also showed no significant pathological changes after 6 weeks of high-salt feeding (Fig. 1b), although the central vein of the hepatic lobule in the high-salt diet group was enlarged. These results indicated that a high-salt diet, on the surface, did not cause significant physical damage to the liver in this timeframe.

### Gross changes in RNA expression in liver tissue with a high-salt diet

**Fig. 2:**
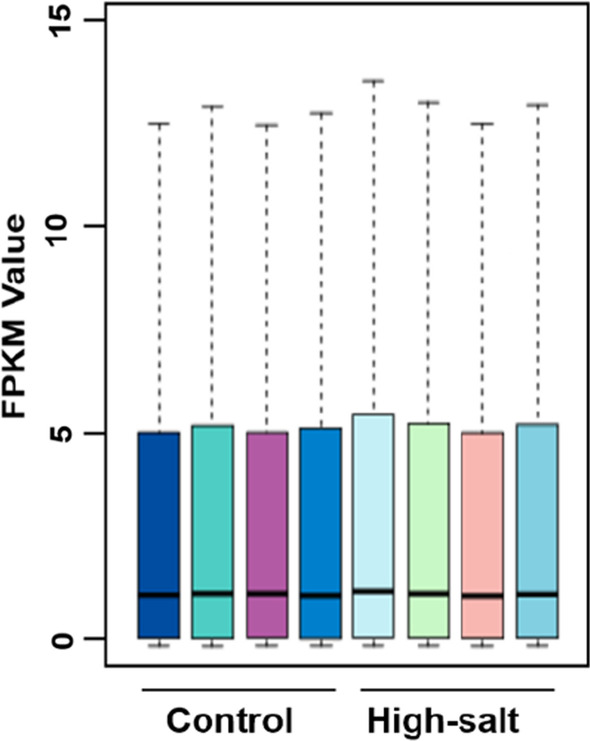
Overall distribution of gene expression in each sample. The expression of each sample was analyzed and is shown with a box diagram. The FPKM value indicates the logarithm of the gene expression from each sample. The bars indicate the range from maximum to minimum. The column indicates the range from upper quartile to lower quartile. The line in each column indicates the median

Although we did not find clear evidence of the effect of a high-salt diet on the liver at the macroscopic level, we hypothesized that the potential effects of this diet on the liver may be subtler. Therefore, we extracted RNA from the livers of mice raised for six weeks with a normal or high-salt diet and used transcriptome sequencing to further analyze the effects of a high-salt diet on liver. After sequencing, 6 Gb of data from each sample was obtained, and after filtering, the high-quality sequences obtained from the normal and high-salt groups were on average 46.02 Mb and 44.68 Mb reads, respectively. The matching rates to a reference genome were 97.43% and 97.44%, respectively, and the total number of genes detected was 18,916 and 19,204, respectively (Additional file 1: Table S1). In order to evaluate the overall trend of sample expression more objectively, we converted the fragments from each gene to Fragments Per Kilobase per Million (FPKM) mapped fragments. Since the number of differentially expressed genes only accounts for a small part of the overall number of genes, and a small number of differentially expressed genes had no significant effect on the expression distribution in our samples, the overall distribution of these samples using a box diagram was virtually the same when we compared each group (Fig. 2). These results indicated that our data met requirements in quality and could be used for further analyses.

### Identification of liver differential genes by GO and KEGG enrichment

**Fig. 3:**
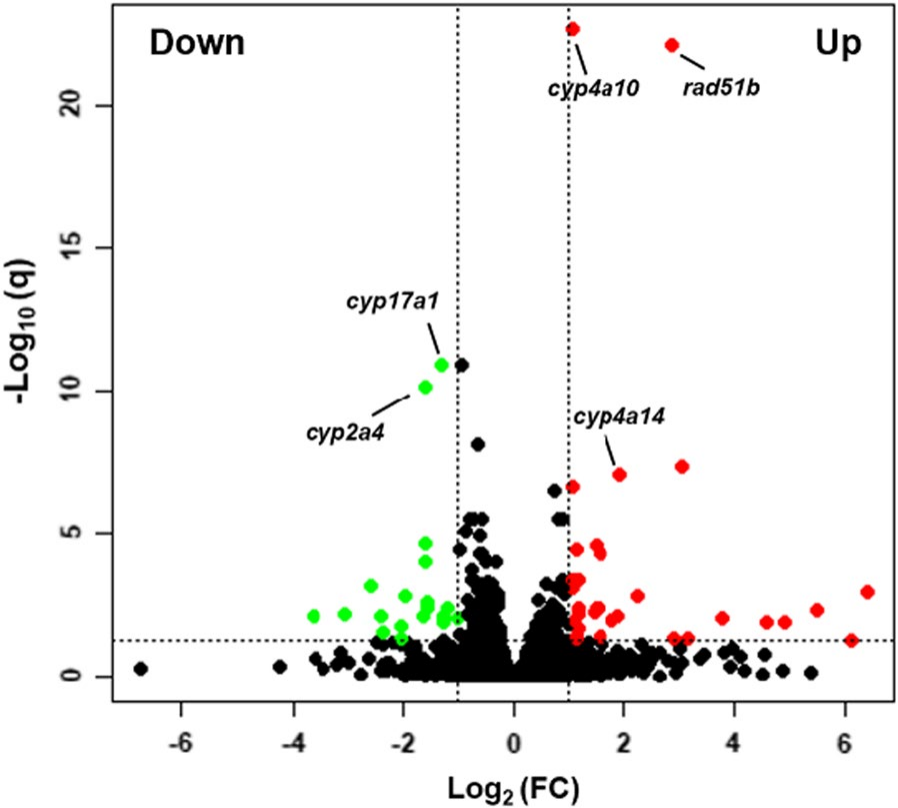
Genes differentially expressed by a high-salt diet. The expressed genes were compared between normal and high-salt diet group, and the differences in the gene expression levels and statistical significance between these two groups are further illustrated by volcano plot. FC indicates the fold change compared with the control group. The green points indicate down-regulated genes, and red points indicate up-regulated genes

During the identification of differentially expressed genes, a fold change ≥ 2 and a padj value (corrected *p* value) < 0.05 were used as screening criteria, and we found that there were 52 differential genes, including 33 up-regulated genes and 19 down-regulated genes. For example, some up-regulated genes like *cyp4a10* and *cyp4a14*, which are related to the formation of non-alcoholic fatty liver (NAFLD), and *rad51b*, which is related to DNA repair, and some down-regulated genes like *cyp17a1*, which is involved in the synthesis of steroid hormones, and *cyp2a4*, which is involved in the metabolism of many drugs and compounds, made logical sense based on previous literature (Fig. 3).

**Fig. 4:**
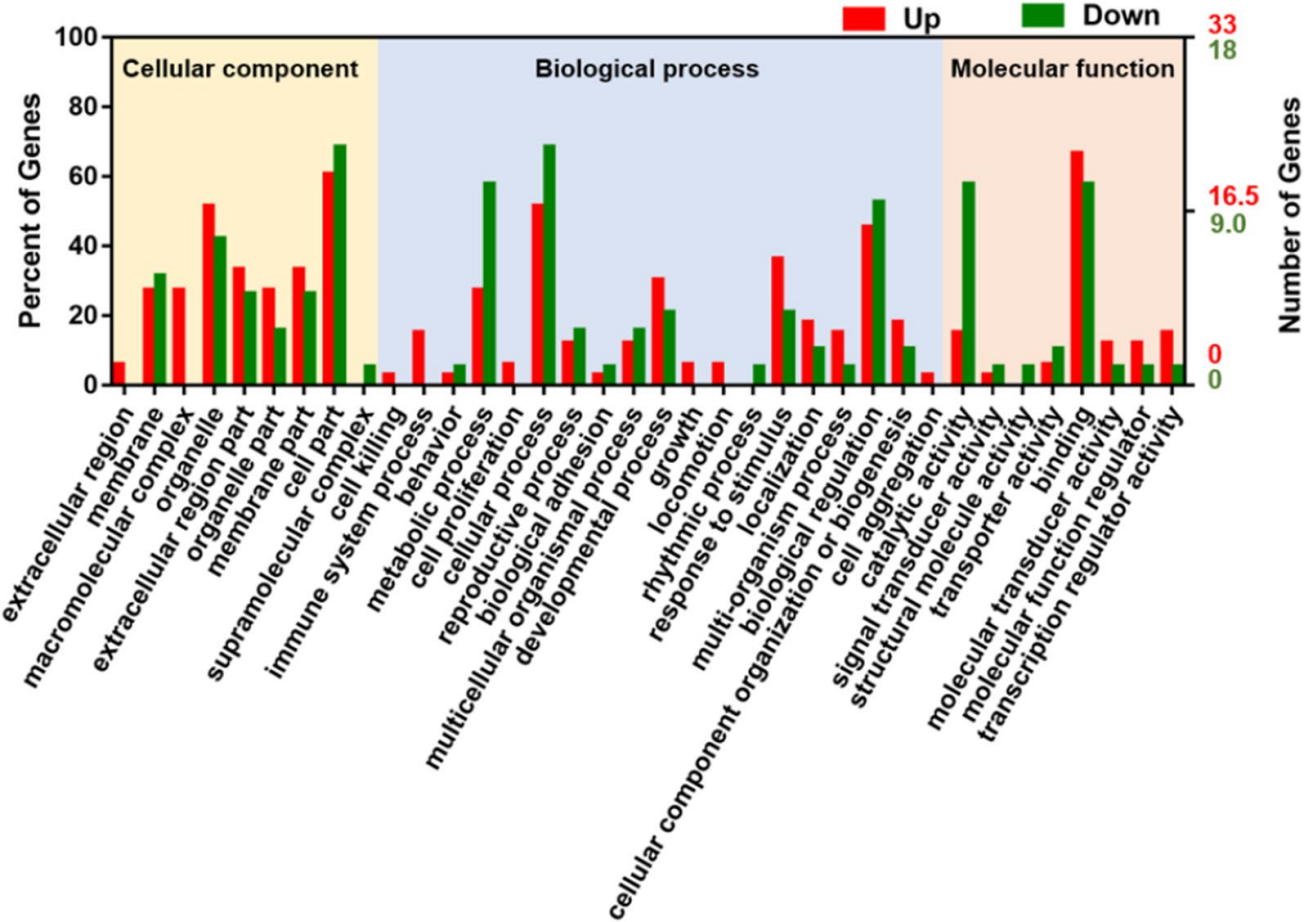
GO enrichment of high-salt diet-induced differentially expressed genes. GO enrichment analysis of differentially expressed genes was performed, and the differentially expressed genes were enriched in 36 sections of the 3 main GO categories (biological process, cellular component, and molecular function). Up indicates up-regulated terms, and down indicates down-regulated terms

In order to further analyze the function of these differentially expressed genes, Gene ontology (GO) analysis was performed. According to the secondary items in the GO database, the number of differentially expressed genes in these items was counted, and percentages were calculated. The results showed that in the 36 sections of the three main GO categories (biological process, cellular component and molecular function), extracellular protein, macromolecular complex, cell killing, immune process, cell proliferation, cell growth, and cell movement were all significantly up-regulated in the high-salt diet group (Fig. 4).

**Fig. 5:**
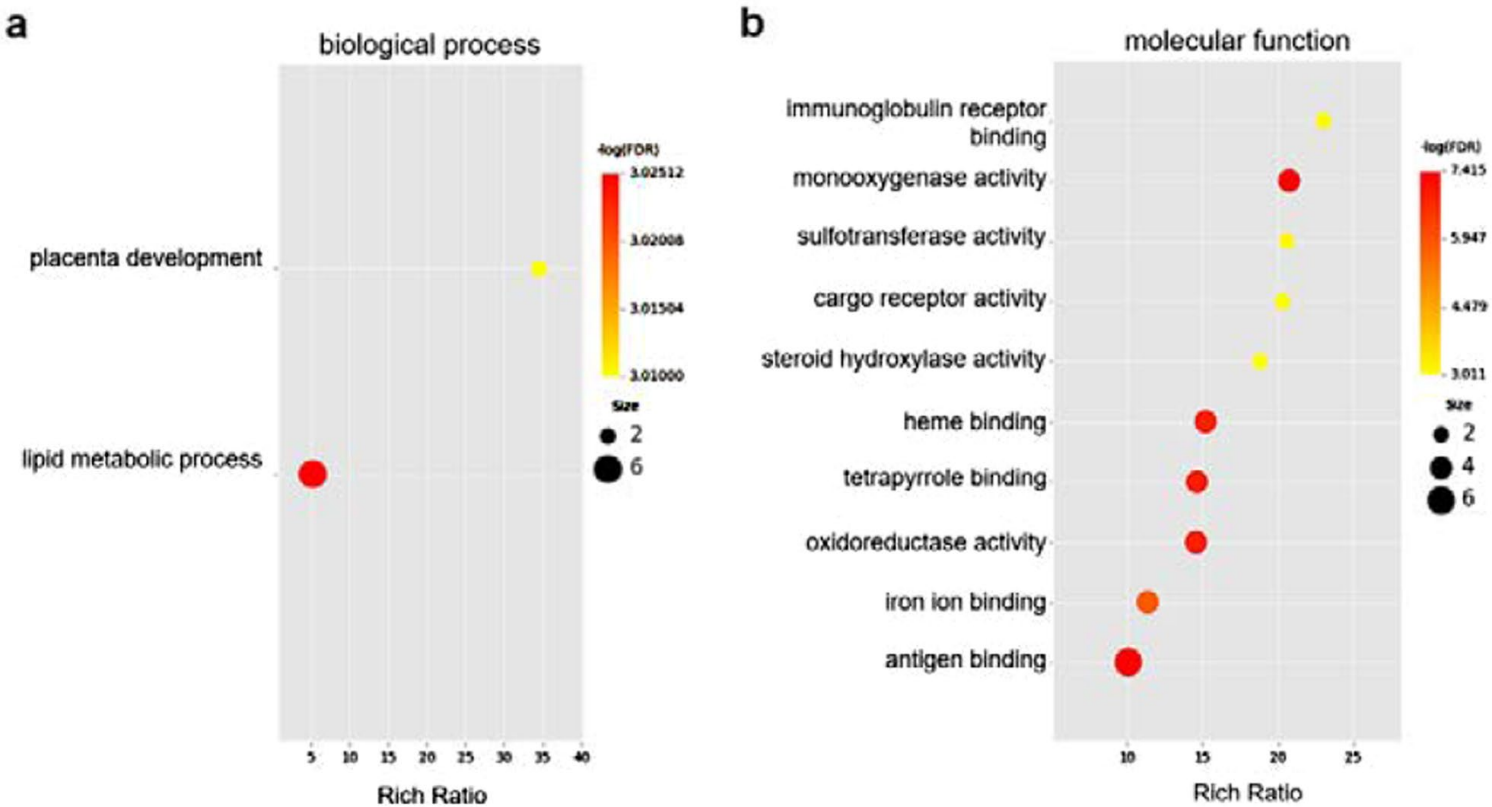
GO enrichment of each category: **a** enrichment in biological process; **b** top 10 enrichment terms in molecular function

On the basis of the differentially expressed genes in each GO entry, we further analyzed these entries in terms of significantly enriched differentially expressed genes compared with a whole genome background. The results yielded 25 significantly different terms, including 2 terms in the biological process category (Fig. 5a) and 23 terms in the molecular function category (Fig. 5b, Additional file 1: Table S2). Specifically, we found that at least 15 enzymatic activities and the metabolism of multiple substances were correlated with a high-salt diet. The abnormal activities of these enzymes and metabolisms have been associated with many diseases.

Next, Kyoto encyclopedia of genes and genomes (KEGG) enrichment analysis showed that the differentially expressed genes induced by high-salt diet were involved in 33 pathways, including some previously reported pathways, such as the peroxisome proliferators-activated receptor (PPAR) signaling pathway [23, 24], the mitogen-activated protein kinase (MAPK) signaling pathway [25], and the prolactin signaling pathway [26]. Some of these had not yet been reported, such as the phosphatidylinositol 3′-kinase—protein kinase B (PI3K-Akt) signaling pathway, retinol metabolism, and steroid hormone biosynthesis (Table 1). These pathways represent potential risks for some diseases.

**Table 1:** KEGG analysis of differentially expressed genes between the high-salt and normal groups

KEGG pathways	KEGG map number	*p*
PPAR signaling pathway	map03320	0.0087
MAPK signaling pathway	map04010	0.3900
Prolactin signaling pathway	map04917	0.1117
PI3K-Akt signaling pathway	map04151	0.5366
TGF-beta signaling pathway	map04350	0.0095
Inflammatory mediator regulation of TRP channels	map04750	0.0182
Signaling pathways regulating pluripotency of stem cells	map04550	0.0210
Aldosterone synthesis and secretion	map04925	0.1241
Metabolism of xenobiotics by cytochrome P450	map00980	0.0083
Drug metabolism—cytochrome P450	map00982	0.1196
Drug metabolism—other enzymes	map00983	0.0980
Linoleic acid metabolism	map00591	0.0876
Ascorbate and aldarate metabolism	map00053	0.0435
Porphyrin and chlorophyll metabolism	map00860	0.0604
Tryptophan metabolism	map00380	0.0616
Retinol metabolism	map00830	0.0000
Arachidonic acid metabolism	map00590	0.0106
Steroid hormone biosynthesis	map00140	0.0004
Ovarian steroidogenesis	map04913	0.0980
Pentose and glucuronate interconversions	map00040	0.0864
Fatty acid degradation	map00071	0.0031
Ubiquitin mediated proteolysis	map04120	0.4150
Endocytosis	map04144	0.4456
Vascular smooth muscle contraction	map04270	0.0184
Bile secretion	map04976	0.0060
Salivary secretion	map04970	0.1083
Homologous recombination	map03440	0.0688
Epstein-Barr virus infection	map05169	0.3673
Peroxisome	map04146	0.1331
Phagosome	map04145	0.2999
Tight junction	map04530	0.3439
Transcriptional misregulation in cancer	map05202	0.3035
Chemical carcinogenesis	map05204	0.0007

## Discussion

As the largest metabolic organ, the liver plays an important role in the health of the body, regulating three major nutrients and various molecular metabolic events, and this organ hosts some key signaling pathway nodes. Thus, abnormalities can have potential impacts on human health. We compared the transcriptome changes in the liver of mice fed with high-salt diet by transcriptome sequencing and found that there were many abnormal signal pathways and metabolic events in the liver after exposure to a high-salt diet. For example, two up-regulated genes, *cyp4a10* and *cyp4a14*, were shown to be involved in the PPAR signaling pathway and related to oxidative stress and lipid peroxidation of fatty acids, causing NAFLD/steatohepatitis (NASH). In steroid hormone biosynthesis pathways, the *cyp17a1* gene was down-regulated, and in the cell proliferation and apoptosis regulation pathways, *nr4a1* gene expression was down-regulated. Many of the pathways we identified here have been previously reported. In addition, we also found some other potential effects of a high-salt diet, such as retinol metabolism, ascorbate and aldarate metabolism, and steroid hormone biosynthesis, which have not been reported previously and can provide mechanistic support for disease prediction of those diseases caused by a high-salt diet.

It has been reported that a high salt diet can cause liver injury by producing excessive reactive oxygen species (ROS), via Nrf2/Keap1 signaling [16]. Although we did not find significant changes in gene expression related to this signaling pathway in our results, which may have been due to differences in the animals and feeding methods used herein, we found that the expression of nicotinamide adenine dinucleotide phosphate (NADPH) oxidase 4, another ROS activating protein, was up-regulated. This also indicated the activation of ROS by a high-salt diet. In addition, many studies have found that a high salt diet was associated with NAFLD [27, 28]. The main mechanism of NAFLD formation has been shown to involve insulin resistance, oxidative stress, and lipid peroxidation, etc. [29]. The role of P450 in this aspect has been consistently demonstrated by others. In addition to the *cyp4a10* and *cyp4a14* genes that may cause NAFLD as mentioned above, we also found the high expression of *cyp2e1*, which can promote oxidative stress and lipid peroxidation, and lead to hepatocyte damage [30–32], accelerating the progress of NAFLD and has been reported in rats and clinical patient studies [33, 34]. Moreover, the expression of *cyp1a2* was shown to be down regulated in NAFLD rats [35], which was similar to our results in high-salt diet fed mice. These results suggest that a high-salt diet may promote NAFLD development by affecting the expression of Cyp protein. In addition, a study also found that the prevalence of NAFLD increased with increase in Na^+^ intake, implying the effects of a high-salt diet on liver lipid metabolism and the potential relationship between NAFLD and obesity could be due to Na^+^ levels [36]. Accordingly, studies have shown that a high-salt intake increases liver osmotic pressure, promotes the expression of the transcription factor TonEBP, and then activates the expression of aldose reductase, promoting the production of endogenous fructose. Therefore, salt may be a potential cause of obesity and metabolic syndrome [15]. Although our results showed that biochemical and H&E staining were normal after 6 weeks of a high-salt diet, indicating no obvious liver damage (probably due to the short time of high-salt feeding, where organic damage had not yet formed), sequencing results indicated the potential influence of this diet on the liver, which needs to be further studied.

There is a significant correlation between high-salt diet and hypertension. A large amount of evidence has shown that salt is the main cause of blood pressure elevation, and a decrease in salt intake reduces blood pressure, thus reducing the diseases related to blood pressure. The central mechanism of hypertension shows that a high-salt diet leads to an increase of the brain’s sodium ion content, which activates the sympathetic nerves through the Na(+)-epithelial sodium channel-renin angiotensin aldosterone system-endogenous digitalis-like factor (Na(+)-ENaC-RAAS-EDLF) axis and promotes the formation of hypertension [37]. It has also been found that salt may induce salt-sensitive hypertension by inhibiting the expression of renal enzymes [38]. In addition, high sodium levels can directly promote the proliferation of vascular smooth muscle cells and promote the formation of hypertension [39]. Our results also showed that the expression of the *cyp4a* gene in the liver was significantly increased, and Cyp4a can hydroxylate arachidonic acid into 20-hydroxyeicosapentaenoic acid and act on blood vessels, which indirectly could participate in the formation of hypertension. This finding also supports a previously established mechanism of high-salt diet leading to hypertension [40].

Although continuous high-salt feeding was only for 6 weeks in our study, there were many metabolic pathways abnormalities that show early hints of liver injury. However, due to the compensation and self-healing ability of liver, it was not clear whether these abnormal could lead to substantial damage in the future. Studies have clearly revealed that high-salt diet is closely related to hypertension, nonalcoholic fatty liver disease, immune abnormalities, etc., and most of the mechanisms driving this are still unknown. Thus, on the basis of this early-stage research, a longer period of study is needed to reveal the dynamic changes in gene expression induced by a high-salt diet. For example, central vein dilatation is the first positive change observed in this study, and is involved in a variety of diseases, such as right-sided heart failure. Further exploration of related genes is necessary and could be achieved by using different doses of NaCl or longer feeding durations. In addition, at present, this early exploration on the effects of a high-salt diet on the liver from a macro perspective also suggests that some other points, like oxidative stress and substance metabolism, should areas of additional research.

As is well-known, the use of male mice is preferrable to the use of female mice, as they have a more accentuated hormone interference. As our original design intent was to explore the changes (including tissue lesions, liver cell abnormalities, inflammation, metabolic abnormalities, etc.) of the liver from the perspective of transcriptomics, the gender of the mice used was not a main factor. In our results, we found that high-salt diet had extensive influence on the liver, especially on liver metabolism. Therefore, this male mouse model can be used for further study.

With the continuous development and popularization of omics research technology (including proteome, transcriptome, and metabolomic) over the past few years, people have gained the ability to systematically describe and analyze the changes of specific levels of the body as a whole, so as to further explore the pathogenesis of diseases. This has allowed them to identify the potential pathogenic risks and influencing factors for many diseases. Among these technologies, transcriptome sequencing technology is an important means to study the pathogenesis of many diseases. Since transcription levels are often positively correlated with protein expression levels, transcriptome sequencing can be used to analyze and predict the occurrence and development of many diseases. By comparing and analyzing the differentially expressed genes under different conditions we can identify changes that may be related to diseases. We also can use this technology to analyze the correlation between various pathways, thus providing clues to the pathogenesis of diseases. This study systematically analyzed the liver after high-salt diet at the transcriptomic level, and we found many potential risks for diseases, which provide clues to the pathogenesis, prevention, and potential drug targets of liver diseases. These potential pathogenic factors will be explored in future research.

## Conclusions

Our study has indicated that a high-salt diet has many potential effects on the liver at the transcriptional level. Although substantial damage has not yet been shown, we found that a high-salt diet can influence at least 15 enzymatic activities and the metabolism of multiple substances. In addition, except for some pathways consistent with known mechanisms of damage caused by a high-salt diet, we also found that a high-salt diet had an impact on other important pathways in the liver. This study, to our knowledge, was the first to reveal the impact of a high-salt diet on the liver at the omics level and provides theoretical support for potential clinical risk evaluation, pathogenic mechanisms, and drug design for the treatment of liver dysfunction. Furthermore, it illustrates a serious candidate direction for the study of the impact of high-salt diets.

## Supplementary Information

**Additional file 1: Table S1.** Comparison of liver transcriptome sequencing data between normal and high-salt conditions. **Table S2.** Enrichment terms in molecular function.

## Data Availability

The datasets generated and/or analyzed during the current study are available in the GEO DataSets, https://www.ncbi.nlm.nih.gov/geo/query/acc.cgi?acc=GSE163294.

## Abbreviations

ALT: Alanine aminotransferase activity
AST: Glutamic oxaloacetic transaminase activity
ALP: Alkaline phosphatase activity
FPKM: Fragments Per Kilobase per Million
GO: Gene ontology
H&E staining: Hematoxylin–Eosin staining
KEGG: Kyoto encyclopedia of genes and genomes
MAPK: Mitogen-activated protein kinase
NADPH: Nicotinamide adenine dinucleotide phosphate
NAFLD: Non-alcoholic fatty liver
Na(+)-ENaC-RAAS-EDLF: Na(+)-epithelial sodium channel-renin angiotensin aldosterone system-endogenous digitalis-like factor
PCR: Polymerase chain reaction
PI3K-Akt: Phosphatidylinositol 3′-kinase-protein kinase B
PPAR: Peroxisome proliferator-activated receptor
ROS: Reactive oxygen species
