# Supplementary material for: Transcriptome sequencing reveals high-salt diet-induced abnormal liver metabolic pathways in mice
Source: BMC Gastroenterol. 2021 Aug 28;21:335. doi: 10.1186/s12876-021-01912-4 (PMC8397858; doi:10.1186/s12876-021-01912-4)
Supplement: Supplementary file 1 — Additional file 1: Table S1. Comparison of liver transcriptome sequencing data between normal and high-salt conditions. Table S2. Enrichment terms in molecular function. [file 12876_2021_1912_MOESM1_ESM.docx]

## Supplementary Materials

Table S1. Comparison of liver transcriptome sequencing data between normal and high-salt conditions

|  | Normal | High-salt | *t* | *p* |
| --- | --- | --- | --- | --- |
| Total data volume (G) | 6 | 6 |  |  |
| Total clean reads (M) | 46.02 ± 0.69 | 44.68 ± 0.40 | 1.69 | 0.14 |
| Mapping Rate (%) | 97.43 ± 0.11 | 97.44 ± 0.18 | 0.04 | 0.97 |
| Total gene number | 18,916 ± 63.67 | 19,204 ± 155.30 | 1.72 | 0.14 |

Table S2. Enrichment terms in molecular function

| **Accession** | **Term_name** | **p-value** | **FDR** | **Rich_Ratio** |
| --- | --- | --- | --- | --- |
| GO:0003823 | antigen binding | 5.50E-06 | 0.000602 | 10.05887083 |
| GO:0004497 | monooxygenase activity | 4.27E-06 | 0.000602 | 20.74352395 |
| GO:0046906 | tetrapyrrole binding | 2.32E-05 | 0.001034 | 14.61475551 |
| GO:0020037 | heme binding | 1.93E-05 | 0.001034 | 15.19013958 |
| GO:0016705 | oxidoreductase activity | 2.36E-05 | 0.001034 | 14.55960549 |
| GO:0005506 | iron ion binding | 7.71E-05 | 0.002813 | 11.34792781 |
| GO:0004092 | carnitine O-acetyltransferase activity | 0.001296 | 0.0258 | 771.6590909 |
| GO:0004304 | estrone sulfotransferase activity | 0.001296 | 0.0258 | 771.6590909 |
| GO:0004508 | steroid 17-alpha-monooxygenase activity | 0.001296 | 0.0258 | 771.6590909 |
| GO:0047442 | 17-alpha-hydroxyprogesterone aldolase activity | 0.001296 | 0.0258 | 771.6590909 |
| GO:0047894 | flavonol 3-sulfotransferase activity | 0.001296 | 0.0258 | 771.6590909 |
| GO:0001760 | aminocarboxymuconate-semialdehyde decarboxylase activity | 0.00259 | 0.040518 | 385.8295455 |
| GO:0005044 | scavenger receptor activity | 0.00225 | 0.040518 | 28.57996633 |
| GO:0015020 | glucuronosyltransferase activity | 0.002503 | 0.040518 | 27.07575758 |
| GO:0034987 | immunoglobulin receptor binding | 0.00344 | 0.044313 | 23.03459973 |
| GO:0008392 | arachidonic acid epoxygenase activity | 0.003241 | 0.044313 | 23.74335664 |
| GO:0008391 | arachidonic acid monooxygenase activity | 0.00334 | 0.044313 | 23.38360882 |
| GO:0035375 | zymogen binding | 0.003883 | 0.044754 | 257.219697 |
| GO:0008389 | coumarin 7-hydroxylase activity | 0.003883 | 0.044754 | 257.219697 |
| GO:0038024 | cargo receptor activity | 0.004401 | 0.0459 | 20.30681818 |
| GO:0008146 | sulfotransferase activity | 0.004289 | 0.0459 | 20.57757576 |
| GO:0008395 | steroid hydroxylase activity | 0.005104 | 0.049264 | 18.82095344 |
| GO:0050294 | steroid sulfotransferase activity | 0.005174 | 0.049264 | 192.9147727 |
